# Impact of drug solvents on *C. elegans* pharyngeal pumping

**DOI:** 10.1016/j.toxrep.2021.06.007

**Published:** 2021-06-17

**Authors:** Fernando Calahorro, Lindy Holden-Dye, Vincent O’Connor

**Affiliations:** Biological Sciences, University of Southampton, Southampton, UK

**Keywords:** High-throughput experiments, Drug screening, Dose-response, Bioassay readouts

## Abstract

*Caenorhabditis elegans* provides a multi-cellular model organism for toxicology and drug discovery. These studies usually require solvents such as dimethyl sulfoxide (DMSO), ethanol or acetone as a vehicle. This raises the need to carefully consider whether the chemical vehicles used in these screens are anodyne towards *C. elegans*. Here, we use pharyngeal pumping as a bioassay to assess this. Pharyngeal pumping is a visually scoreable behaviour that is controlled by environmental cues activating sensory and integrative neural signalling to coordinate pharyngeal activity. As such it serves as a rich bioassay to screen for chemical modulation. We found that while pumping was insensitive to high concentrations of the widely used drug solvents ethanol and acetone, it was perturbed by concentrations of DMSO above 0.5 % v/v encompassing concentrations used as drug vehicle. This was manifested as an inhibition of pharyngeal pump rate followed by a slow recovery in the continued presence of the solvent. The inhibition was not observed in a neuroligin mutant, *nlg-1*, consistent with DMSO acting at the level of sensory processing that modulates pumping. We found that *bus-17* mutants, which have enhanced cuticle penetration to drugs are more sensitive to DMSO. The effect of DMSO is accompanied by a progressive morphological disruption in which internal membrane-like structures of varying size accumulate. These internal structures are seen in all three genotypes investigated in this study and likely arise independent of the effects on pharyngeal pumping. Overall, these results highlight sensory signalling and strain dependent vehicle sensitivity. Although we define concentrations at which this can be mitigated, it highlights the need to consider time-dependent vehicle effects when evaluating control responses in *C. elegans* chemical biology.

## Highlights

• DMSO impacts on *C. elegans* feeding behaviour in short-term and in a reversible manner.
• DMSO affects the cellular integrity of *C. elegans*.
• *nlg-1* mutant is refractory to DMSO induced pumping inhibition.
• Judicious consideration of the effect of the DMSO in bioassays in *C. elegans*.

## Graphical abstract

**Figure:**
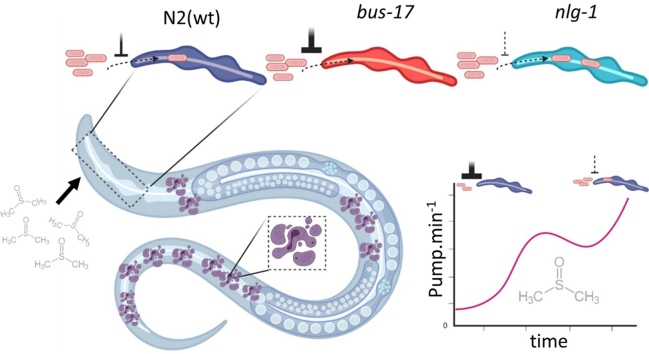

## 1 Introduction

One approach to developing robust experimental systems to define drug mode of action and/or toxicology is through the use of simple model organisms [[1], [2], [3]]. This approach represents an intermediary between *in vitro* and *in vivo* platforms. *Caenorhadbitis elegans* is an attractive model organism for medium and high-throughput screening. Among the advantages one can highlight its easy manipulation, a short life cycle, low-cost and accessibility to whole organism imaging. This experimental tractability is further supported by its genetic tractability, significant conservation of the proteome and genetic pathways with humans [[4], [5], [6]]. *C. elegans* is a particularly suitable model for approaches where genetic manipulations need to be combined with pharmacological studies. This flexibility has led to discovery platforms and mode of action investigations for nematicides, compounds with anti-aging properties as well as a range of therapeutic interventions [[7], [8], [9]].

The employment of model organisms in high-throughput screening is viewed as a promising strategy to identify genes or drugs potentially relevant to health and fitness as well as to target key determinants in disease. In particular, the ability to generate large numbers of animals allied to high throughput observational measurement lends itself to the development of whole organism bioassays [[10], [11], [12], [13], [14]]. In the context of *C. elegans,* motility and to some extent feeding behaviour, have been successfully used strategies [[15], [16], [17]].

In the case of *C. elegans* feeding behaviour the ability to rapidly present and remove bacteria allows the investigator to trigger and readily score increases in pharyngeal pumping. The response reflects a sensory detection and downstream neuronal signalling to execute an increase in the frequency of the contraction-relaxion cycle that can be visually observed or recorded in the intact worm [18,19]. This provides a quantitative readout that is underpinned by a range of transmitter pathways and embedded molecular targets providing an excellent model system *per se* for drug screening and has been used to identify receptor targets. More widely the pharyngeal system can be combined with the intrinsic genetic tractability to heterologously express drug targets from a range of different species including humans in which the bioassay embedded in pharyngeal pumping can be used to investigate the expressed drug target [20]. Finally, the pharynx is a sensitive biosensor that can provide a sub-lethal readout of drug toxicity [21,17]. This all evidences the value of the pharyngeal system as an important platform of drug investigation in the intact worm [22,23].

A confound of pharmacological approaches in the intact organism is control of drug access to the internal tissues and organs of the worm due to the cuticle. The presence of this surrounding exoskeleton can make drug access more difficult to predict [[24], [25], [26]]. However, mutations that increase cuticular permeability and/or judicious utilization of suitable drug vehicle can help overcome these limitations [2].

Chemical compound screens are regularly performed in popular amphipathic solvents like dimethyl sulfoxide (DMSO) or dimethyl formamide (DMF). However, studies identify differential interference of the biological assays by distinct classes of drug solvent. In the case of DMSO, which is the stock solvent in many industrial and commercial libraries *per se* effects on *C. elegans* physiology and homeostasis are documented. These solvent effects include effects on fertility, lifespan or feeding mechanisms [[27], [28], [29], [30], [31], [32], [33], [34]].

In this study we use Bristol N2 (wild-type), *bus-17* and *nlg-1 C. elegans* strains to test the effect of the widely used drug solvents DMSO, ethanol and acetone. *bus-17* is one of a class of genes (*bus-2, bus-4, bus-12, srf-3, bus-8*) that encode proteins that impact on the composition of the worm cuticle which when mutated increase worm permeability to drugs [35]. We chose pharyngeal pumping as an integrated bioassay in *C. elegans*. We show that DMSO has an effect on the pharyngeal activity in the wild type N2 strain, leading to inhibition of the pharyngeal pumping in a short-term. This effect was recovered ∼3 h after initial exposure in the continued presence of DMSO and was not seen in *nlg-1* mutants that are known to control sensory processing of environmental cues [36]. In contrast this inhibition appeared more pronounced in *bus-17* mutants, a genetic background used to sensitize bioassays to drug exposure. Finally, and independent of the modulation of the expression of sensory effects of DMSO and its modulation by cuticle integrity, we describe morphological changes of internal body composition following prolonged exposure to DMSO. These effects arise over a time course that would be encountered in some drug screening paradigms. Overall, this highlights the need to analyse an underling modulatory effect of organic solvents at concentrations that might be otherwise well tolerated in *in vitro* assays of drug action. Thus, when using simple drug exposure strategies in whole organism bioassays of *C. elegans* these confounding vehicle effects should be considered and controlled for.

## 2 Methods

### 2.1 Culturing of *C. elegans*

Wild-type, Bristol N2, the mutant strains *nlg-1 (ok259) X* and *bus-17* (*e2800*) *X*, were obtained from the *Caenorhabditis elegans* Genetic Centre (University of Minnesota, Minneapolis, MN, USA). *nlg-1* and *bus-17* strains were backcrossed 6 and 3 times respectively with Bristol N2 strain. All strains were grown and maintained at 20 °C under standard conditions [50]. Worm age was synchronized by picking L4 larval stage animals to new plates 18 h prior to performing the behavioral and/or imaging experiments.

### 2.2 Making assay plates

50 μl, 5 μl or 2.5 μl volumes of 100 % analytical grade DMSO, ethanol or acetone were soaked into 5 ml of NGM-medium of individual wells of a 6-well plate. This gave desired final concentrations of each solvent: 1 %, 0.75 %, 0.5 %, 0.25 %, 0.1 % and 0.05 % (V/V). This is equivalent to the following mM predicted concentrations of each solvent: DMSO – 141 mM, 105.75 mM, 70.5 mM, 35.25 mM, 14 mM, 7 mM; ethanol – 171 mM, 128.25 mM, 85.5 mM, 42.75 mM, 17 mM, 8.5 mM; acetone – 136 mM, 13.5 mM, 6.7 mM.

**Fig. 1:**
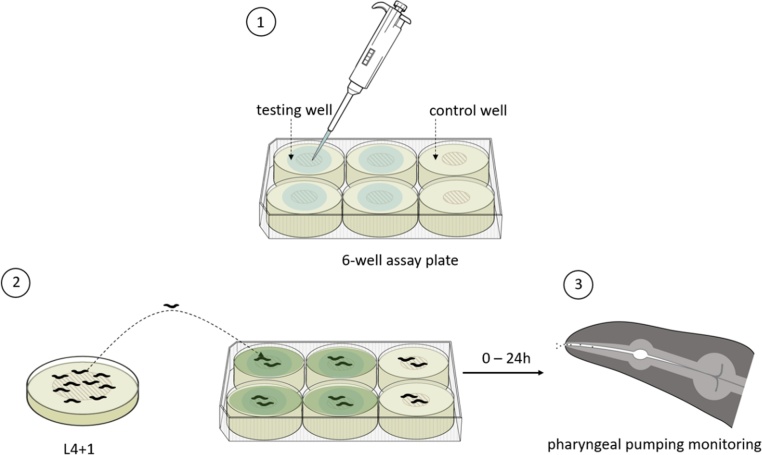
**Experimental design used to test the effect of solvents on pharyngeal pumping.** Volumes of DMSO, ethanol and acetone were soaked into NGM-medium to reach the desired v/v (%) concentrations (0.05–1 %). An *E. coli* OP50 lawn was created in the center of individual wells using a volume of 50 μl of bacterial culture. Control wells without any solvent were included within individual plates as an untreated condition reference. (1). After solvent equilibration into testing wells (represented by light green shadow) 1-day-old adult worms (L4 + 1) grown at 20 °C were picked onto the middle of the *E. coli* OP50 bacterial lawn (2). 10 min after picking, the pharyngeal pumping of individual animals was monitored at indicated times (3).

After adding the desired volumes of solvents, plates were immediately sealed and left for 12 h at RT to allow equilibration of the solvent into wells, as previously described [43]. Then 50 μl of *E. coli* OP50 (OD_600_ of 0.8 AU) were deposited on top of the vehicle laced agar and incubated for a further 12 h at RT (≈20−23°C) (Fig. 1).

### 2.3 Measuring pharyngeal pumping activity

Pharyngeal activity was scored by measuring frequency of pharyngeal pumping, where a single pharyngeal pump is one contraction-relaxation cycle of the terminal bulb of the pharyngeal muscle. This was visually scored by counting the number of pharyngeal pumps for 1 min using a binocular dissecting microscope (Nikon SMZ800; ×63). 1-day-old adult worms (L4 + 1) grown at 20 °C were picked onto the middle of the bacterial lawn (OD_600_ of 0.8 AU seeded the day before), where they were exposed to solvent for up to 24 h. After 10 min (time 0) the pharyngeal pumping was recorded using a hand counter. Five consecutive measurements (1 min each) at each time point were made and the mean of pharyngeal pumping rate was calculated. Pumping was monitored at the time-points: 0 min (10 min. post picking to food laced observation place with and without vehicles), 15 min, 30 min, 1 h, 3 h, 4 h, 5 h, 6 h and 24 h.

### 2.4 Imaging worms to assess motility posture and integrity and appearance of internal compartments

After the indicated time of exposure to solvents in solid agar plates, the whole worm posture (loss of naïve body bends against straight or coiled forms) was observed and imaged. During these observations we noted the apparent disruption in the appearance of the worm’s internal structures. This included the accumulation of internal membrane-like structure. These structures were captured by imaging with a Nikon Eclipse X. After incubation on untreated or solvent containing agar the worms were transferred into 0.5 μL M9 buffer on a thin 2% agarose pad containing sodium azide (10 mM).

These mounted worms were then covered with a 24 × 24 mm cover slip and were observed with a 40–63X objective magnification for no more than 10 min after being placed onto the sodium azide agarose pad. At least 5 independent worms per condition were analyzed and imaged. Images were captured through a Hamamatsu Photonics camera and acquired using IC Capture 2.2 software and composed with Abode PhotoShop® (Adobe Systems) and ImageJ (NIH) softwares.

### 2.5 Statistical analysis

Statistical analyses were made using GraphPad Prism software. Data are expressed as means ± s.e.m. The statistical tests and post-hoc analyses are indicated individually in graphs.

## 3 Results

### 3.1 DMSO impacts on *C. elegans* feeding behaviour in short-term and in a reversible manner

**Fig. 2:**
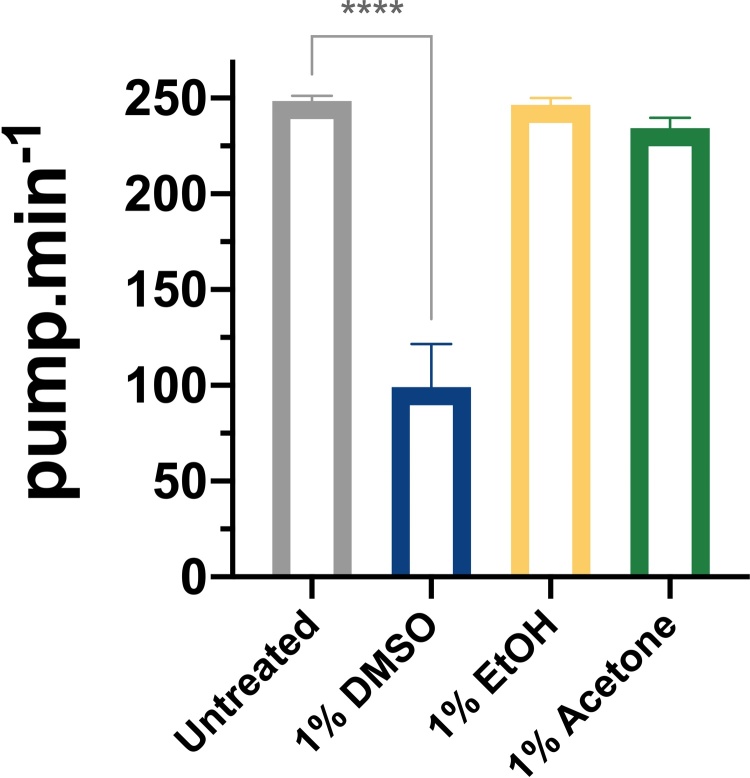
**DMSO inhibits pharyngeal pumping in an acute exposure.** N2 (wt) worms were picked onto both untreated and drug treated plates and pharyngeal pumping was monitored 10 min. after being introduced into this arena. Acute exposure to DMSO leads to a significant reduction in the pharyngeal pumping rate compared to untreated worms (**** p ≤ 0.0001; n = 5). Data represent the mean ± s.e.m of pharyngeal pumping rate. The means were calculated from data collected from repeats of the same experiment conducted on different days. Statistical analysis was performed using one-way ANOVA (Bonferroni multiple comparisons test).

To determine the effect of the solvents DMSO, ethanol and acetone on feeding behaviour we monitored the pharyngeal pumping over time in the wild type Bristol N2 strain. First, we measured the frequency of pumping in the presence of each solvent (1%) relative to untreated conditions (Fig. 1). We did not observe any effects on the bacterial lawns in control and solvent loaded plates. We observed a strong reduction of the pharyngeal pumping rate of N2 (wt) worms in presence of 1% (v/v) (141 mM) DMSO after 3 h of exposure to the solvent (Fig. 2). In contrast, we did not find any reduction in pharyngeal pumping when animals feed in the presence of ethanol (1% (v/v); 171 mM) and/or acetone (1% (v/v); 136 mM).

**Fig. 3:**
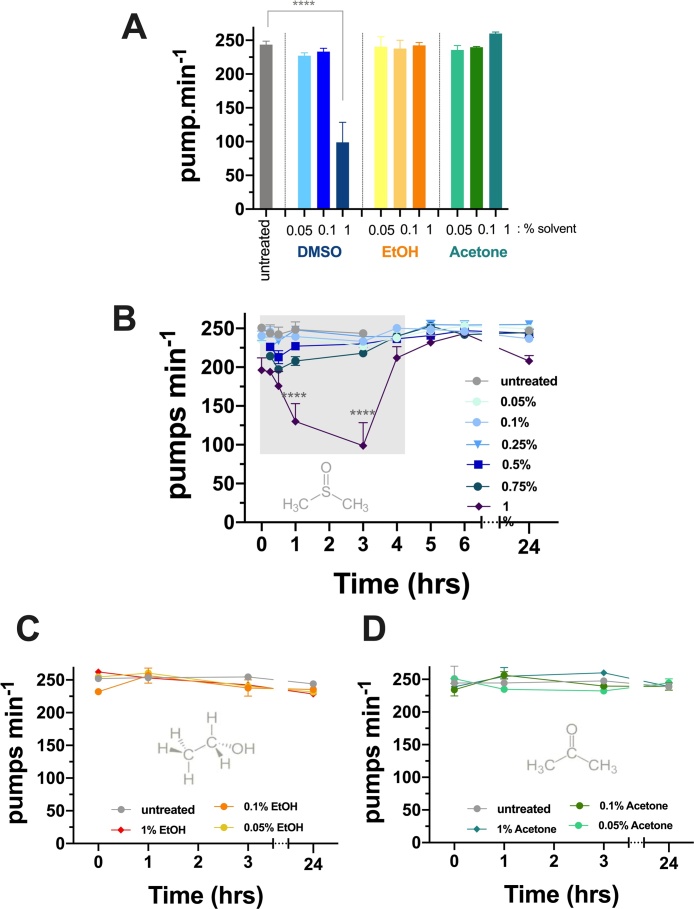
**DMSO induces a rapid and reversible inhibition of pharyngeal pumping.** **A.** After 3 h of acute exposure to increasing concentrations of DMSO, ethanol or acetone (0.05–1 %), only 1% DMSO induces a profound inhibition of pharyngeal pumping (**** p ≤ 0.0001; n = 7). **B.** The chronic exposure at 1% DMSO induces a rapid pumping inhibition in N2 (wt) worms, reaching the maximum level of inhibition (showing with a light grey shadow block on the graph) in a time window of 1−3 hrs (**** p ≤ 0.0001; n = 8). No effect on pharyngeal pumping is observed during ethanol (**C**) or acetone (**D**) exposure. Data represent the mean ± s.e.m of pharyngeal pumping rate. The means were calculated from data collected from repeats of the same experiment conducted on different days. Statistical analysis was performed using two-way ANOVA (Bonferroni multiple comparisons test).

We also studied the effect of solvent on pumping rate at lower concentrations to analyse the threshold of the effect on feeding behaviour. We found that N2 animals feeding on bacteria in the presence of low concentrations of DMSO (0.05 %–0.1 % (v/v); 7−14 mM) do not have a reduction in the pumping rate when compared to untreated animals (Fig. 3A). For the rest of the solvents, no significant differences were observed when comparing the rate of pumping at lower concentrations relative to the highest concentration tested (1% (v/v)) (Fig. 3A).

Next, we monitored the pharyngeal pump rate at increasing times following exposure to solvent. We wanted to analyse what the longer-term exposure to these concentrations of solvents would be by monitoring pumping after 24 h of incubation at the distinct doses. Protracted exposure to 1 % (v/v) ethanol (171 mM) and acetone had no effect on the pump rate when compared to untreated worms. In keeping with the observations made when considering short exposure to DMSO we noted inhibition in the pump rate steadily increased up to a maximal inhibition at the previously investigated 3 h time-point (Fig. 3A). Surprisingly, by 4 h the pumping had recovered to pre-exposure levels and this recovered pumping was maintained for up to 24 h despite worms being continually exposed to 1% v/v DMSO throughout. This indicates a recovery from the inhibition in the face of persistent solvent exposure. For the other DMSO *d*oses, no changes across the time course were observed compared to the untreated group (Fig. 3B).

**Fig. 4:**
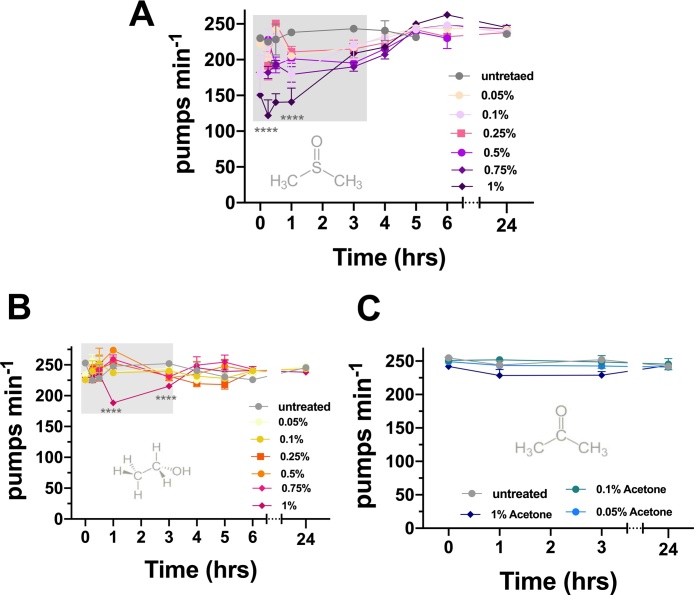
**Ethanol induces a modest and reversible inhibition of pharyngeal pumping in *bus-17* mutants**. *bus-17* deficient-mutants feeding in presence of a chronic incubation with DMSO (0.05–1 %) (**A**), as well as ethanol (1%) (**B**), present a significant reduction over time (showing with a light grey shadow block on the graph) in pumping rate compared to animals in untreated conditions (**** p ≤ 0.0001; n = 5). Acetone does not affect the pharyngeal pumping over time (ns, *P* > 0.05. n=6) (**C**). Data represent the mean ± s.e.m of pharyngeal pumping rate. The means were calculated from data collected from repeats of the same experiment conducted on different days. Statistical analysis was performed using two-way ANOVA (Bonferroni multiple comparisons test).

We also monitored pharyngeal pumping in the presence of these solvents in the *bus-17* mutants. When considering these solvents, acetone showed no impact on the pharyngeal pump in *bus-17*. Surprisingly, we observed an apparent sensitivity to ethanol not observed in N2 (wt) animals (Fig. 4). This is a surprise as our previous experiments suggested that solvents like ethanol readily equilibrate across the cuticle [43]. We also noted a shift in the sensitivity of pumping in the *bus-17* worms. This is highlighted by the DMSO at a concentration of 0.1 % (v/v), which failed to inhibit pumping in the N2 (wt) animals, causing a significant inhibition of pharyngeal pumping (Fig. 4). Although the *bus-17* mutants seemed to be sensitized to the DMSO induced inhibition, pumping recovered to pre-exposure levels after a peak inhibition in the *bus-17* mutants. Thus, as for N2 (wt), pumping recovers in the *bus-17* mutants despite the protracted incubation in the DMSO.

### 3.2 The sensory processing deficient-mutant nlg-1 is refractory to DMSO induced pumping inhibition

**Fig. 5:**
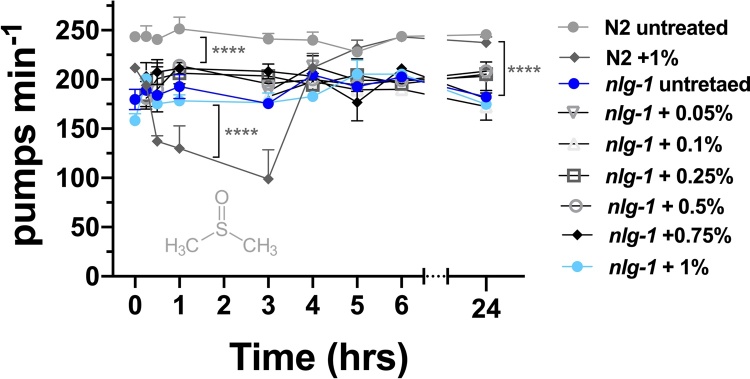
**Neuroligin mutants are refractory to the inhibitory effect of DMSO in pharyngeal pumping**. *nlg-1* mutants feeding in presence of DMSO sustained their pharyngeal pumping, without inhibition over time. There were no significant differences between different concentrations of DMSO at any of the individual time points (ns, *P* > 0.05; n=7−10). Data represent the mean ± s.e.m of pharyngeal pumping rate. The means were calculated from data collected from repeats of the same experiment conducted on different days. Statistical analysis was performed using two-way ANOVA (Bonferroni multiple comparisons test).

We tested the impact of DMSO on the feeding behaviour of the neuroligin-deficient mutant *nlg-1 (ok259)*. These mutants present sensory processing deficits against a wide range of mechanical, chemical and gustatory cues [36,37]. First, we measured the frequency of pharyngeal pumping in *nlg-1* mutants in untreated conditions and we found that *nlg-1* animals have a low frequency of pumping on food compared to wild-type (Fig. 5) [38]. Secondly, we tested the pharyngeal behaviour of *nlg-1* animals incubated with the highest dose (1% (v/v)) of DMSO tested in the previous experiments with N2 (wt) animals. *nlg-1* mutants did not show a decrease of the feeding rate in presence of this dose of DMSO (Fig. 5). These results are consistent with the fact that neuroligin mutants have a deficit between the sensing of environmental cues by sensory neurons, and the processing and integration that generates an aversive response to DMSO.

### 3.3 Chronic exposure to DMSO induces a disruption of normal body posture during locomotion and further morphological changes in the internal structures

During observation of the effects of solvent on pharyngeal function we noticed disruption of the worm’s gross motility and morphology particular to exposures longer than 3 h. To gain further insight we investigated the consequence of exposing worms to this solvent on the anatomical integrity of the treated worms.

**Fig. 6:**
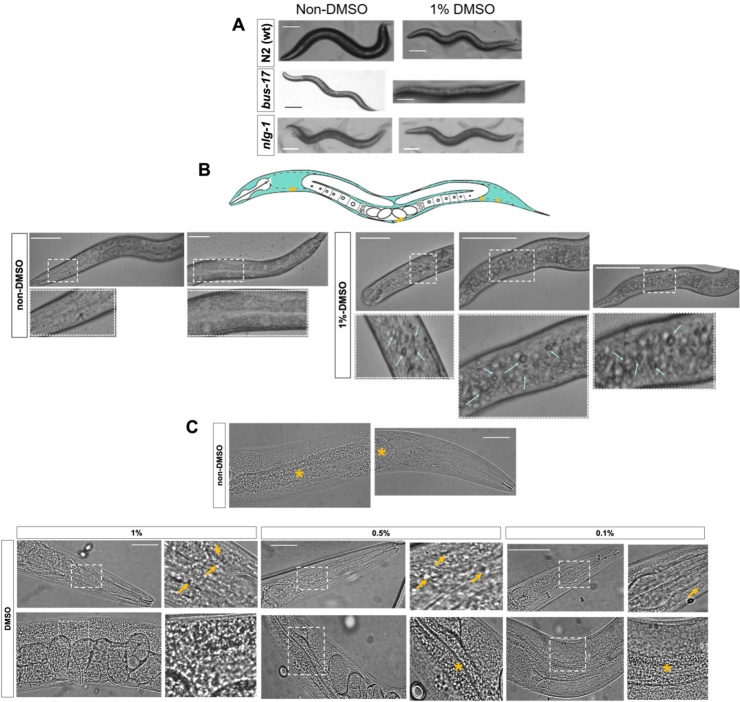
**DMSO compromises the motility posture and morphological integrity of *C. elegans* during a chronic exposure**. **A.***bus-17* animals lack the normal wave shape of the body in a sustained exposure to DMSO at a concentration of 1% compared to the effect on N2 (wt) and *nlg-1* animals. Representative images are shown where a total of 5 independent animals were imaged after 3 h of chronic exposure. The scale bars indicate 100 μm. **B.** At a macro-cellular level, the prolonged DMSO incubation produces an accumulation of internal membrane-like structures within the worm’s body cavity (arrows). The representative images shown correspond to N2 (wt) animals, and the same effect was observed in *bus-17* and *nlg-1* animals (see Supplement to Fig. 6). On top, schematic representation of *C. elegans* anatomy highlighting the pseudocoelom surrounding worm’s cavity (light green), as well as the six coelomocytes cells (yellow dots). The scale bars indicate 130 μm. **C.** Representative images of *bus-17* animals after 3 h of DMSO chronic exposure in increasing concentrations, showing a cumulative appearance of corpuscles (arrows) around the head and middle region of the body. Asterisks indicate intestinal fat. Top a representative image of an untreated animal. A zoomed view showing the accumulation of corpuscles is in the inset box on the right side of each image. The scale bars indicate 100 μm.

We incubated the worms for 3 h, where the maximum inhibition of pharyngeal pumping had been observed, at concentrations between 0.05–1 % of DMSO. Under control conditions the *C. elegans* posture was described by changes to the sinusoidal body’s bends as the worm bends its body during crawling in “S” shapes (Fig. 6A). In contrast this was disrupted in the worms exposed to 1% (v/v) DMSO (Fig. 6A). This was reflected in a flattening of the typical S posture expressed by the worms when moving on food. This flattening during movement on food was more obvious in *bus-17* animals and in some cases the worms lost all undulatory appearance and adopted a straight posture, such postural changes were modest in N2 (wt) or *nlg-1* animals (Fig. 6A). This postural effect was reversed after 24 h when the affected worms were removed to a DMSO free bacterial agar plate, returning to a normal pattern of locomotion. With respect to these observer-based descriptions of motility posture no changes were noted at concentrations lower than 1% DMSO (from 0.1−0.75%).

When observing the shift in worm’s motility we recognized a change in the appearance of the internal organization of the worm under light microscope. At concentration of 1% DMSO, which are used in some drug treatments, we noted that the worms started to accumulate internal membrane-like inclusions. These structures, which are not observed in age matched untreated worms, accumulated along the entire worm’s pseudocoelom surrounding the pericellular (Fig. 6B). We also observed a progressive accumulation of these internal membrane-like inclusions which ranged from between 3 and 10 μm after 1% DMSO *d*osing for 24 h in the N2 (wt), *nlg-1* and *bus-17* mutants (Fig. 6C). They were not observed at the DMSO *d*oses described below 1%. The appearance of these internal membrane-like inclusions was sustained over time with no clearance after 24 h of chronic exposure (Supplement to Fig. 6). The formation of internal membrane-like inclusions appeared selective to DMSO solvent exposure and was not observed in animals after chronic exposure to ethanol or acetone.

## 4 Discussion

Our study highlights the importance of choosing a suitable solvent during experimental approaches based on drug treatments of *C. elegans*. Our analysis was motivated by our attempts to improve throughput for whole organism approaches focussed on using pharyngeal pumping as a bioassay for drug action. We and others have usefully developed this feeding organ as a robust bioassay and convenient approach to investigate the activity and mode of action of an increasing number of compounds [23,38]. Furthermore, the *C. elegans* pharynx offers itself as a suitable platform for heterologous expression of drug target activity to allow it to serve for wider drug screening [20,39]. An underappreciated determinant of drug screening is the vehicle dilution of the solvents that are routinely used to provide multiplexed chemical and drug libraries [[40], [41], [42]]. The widely used solvent DMSO is favoured as it shows good solvation for a wide range of chemical classes and can be well tolerated by screening platforms at concentrations up to and below 1 % [42].

In *C. elegans* the pharyngeal system offers a robust bioassay for whole organism drug testing but is a readout that is dependent on complex cellular and intercellular communications that are additionally susceptible to integrating environmental cues [38,17]. Here, we show that common drug vehicles are well tolerated but identify potential DMSO sensitivity at the higher concentrations at which it is sometimes used as drug vehicle in other systems. Thus, we observe that DMSO interferes with food induced pharyngeal pumping activity of *C*. *elegans* in the N2 (wt) strain. This impact is noted to concentrations above 0.1 % (v/v) which are sometimes used in drug screening approaches. We also found the *C. elegans bus-17* mutant which has a compromised cuticular integrity and is used to sensitize efficacy in whole organism drug screens has an enhanced sensitivity to DMSO [2]. Thus, when using these mutants, one should make a careful consideration of confounding vehicle effects.

It is interesting that there was also a slight sensitization to an inhibition of pharyngeal pumping at the higher concentrations of ethanol tested in this study. Our previous experiments highlighted based on bioassay readouts that ethanol readily crossed the intact cuticle which did not present as a permeability barrier to this solvent [43]. However, the experiments presented here indicate ethanol permeability in the context of food may impact the rate at which ethanol equilibrates into the worm [43,44].

The chemical nature of DMSO allows it to interact with chemicals and provide a shield from water and promote solubility of hydrophobic molecules that are otherwise insoluble in water. Although attractive as a solvent, DMSO can perturb integrity of biological systems. The more dramatic of these effects are usually played out at much higher concentrations than it would be used at in drug vehicle experiments. We observed a significant inhibitory response to DMSO concentrations above 0.1 % (v/v) (14 mM) at pharynx level in both N2 (wt) and *bus-17* mutants. The acute onset of inhibition observed is reversed upon prolonged exposure to the drug. This suggests that sensory processing might be an important determinant of this inhibitory effect. In line with our observations, it has been described that DMSO concentrations between 0.8–1 % induce a reduced brood size, as well as significantly increase lifespan, likely acting through a mechanism dependent on insulin-like signalling. This suggests that there could be a fitness cost for the observed pharmacologically induced long-lived animals [34]. In our bioassays, at that concentration (1%), we observed a severe impact on pharyngeal activity in *C. elegans*, which is in line with the suggestions of an impact of the global fitness state.

We had previously shown that the synaptic protein neuroligin (NLG-1) was an important extrapharyngeal determinant of pharyngeal pumping. *nlg-1* mutants present a reduced pumping that suggests that neuroligin organizes extrapharyngeal circuits that regulate the pharynx, highlighting the role of neuroligin in discretely impacting functional circuits underpinning complex behaviours [38]. Interestingly, *nlg-1* mutants lost their sensitivity to DMSO. This does not rule out important contribution of direct perturbation effects of key cells involved in co-ordinating pharyngeal pumping but does highlight that DMSO at 1% and above concentrations can provide an additional selective modulation of sensory modalities. This could imply interference with sensory detection of food in the neural circuits that regulate pharyngeal activity. This is consistent with sensory processing defects in *nlg-1* previously described [38], as the lack of an aversive response by the normally aversive chemical 1-octanol, or the defects in the processing of two conflicting chemosensory inputs [37].

Interestingly, we observed a response to 1 % (v/v) (141 mM) DMSO at the whole worm level disrupting the *C. elegans* posture typical of untreated worms. These observations showed that DMSO exposed animals, do not likely induce lethal changes in metabolism. However, we did note changes in worm posture from which they recovered and consistent with effects of the solvent on the body wall muscle system that controls motility [49]. In addition, we observe exposure to DMSO resulted in the formation of a disrupted morphology. This coincided with the appearance and accumulation of internal membrane-like structures. These changes seem to be particular to DMSO relative to the solvents tested in this study. These structures appear membranous in structure however at the light microscopic level we cannot resolve if these are double membrane vesicles or single lipid boundaries defined by visible lipid droplets that are present and dynamically change in nematodes [45,46]. Both remain a possibility and the accumulation of DMSO and solvents within the pseudocoelom, following external exposure, would be well placed to trigger signaling and metabolic changes that disrupt cellular and wider sub-compartment integrity. The size and features of the internal membrane-like structures would be compatible with DMSO triggering the accumulation of corpuscular apoptotic cells [47], disrupted and enlarged lipid droplet trafficking [45] or the activation of the recently recognized cellular vacuolization that arises through the processes of methuosis [48]. One speculation is these structures are a stress response and/or metabolic disruption, potentially compromising the intra and/or extra cellular integrity, and that is subject to regulation by the scavenging systems of the worm encompassed in coelomocytes. We observe these changes in the extreme of prolonged DMSO exposure at the upper range at which it is used for drug vehicle. However, the selective effect of DMSO relative to other solvents tested, alerts to potential confounds associated with chronic dosing experiments in which DMSO is the background vehicle. Indeed, their appearance within the worms pseudocoelom and persistence following DMSO removal would be consistent with this idea (Fig. 5B).

In conclusion, here we provide direct evidence for a differential effect of molar equivalents of a common drug solvent DMSO used in the investigation of drug administration in *C. elegans*. These data highlight that the function of the pharynx, the neuromuscular organ that controls feeding is not affected by DMSO up to concentrations of 0.1 %. However, higher concentrations including those that are used for vehicle controls impact on pharyngeal function. Although, we have limited the investigation of these solvents in solid phase in this study, it is worth considering that the effective concentrations of solvents impacting on physiological parameters such as development, fitness or complex behaviour could vary in different media *i.e.* axenic liquid culture. In the current paper we directly monitor pharyngeal pumping as this is feasible in the agar plate format. Higher throughput assays are achieved in liquid culture in which animals are supported by bacteria in solution rather than those plated in solid phase on agar [10,11]. The confounds in which DMSO *d*isrupts feeding as mapped out in this paper are likely to pertain to other experimental formats that utilize DMSO *d*issolved drugs as a central factor to their approach. DMSO while acting as an excellent drug vehicle has known concentration dependent disruptive effects. As shown here, it has the ability to initiate and or modulate sensory modalities in whole organism bioassays. Overall, the findings alert investigators to judicious consideration of both the acute and chronic effect of the common drug vehicle DMSO in bioassays in the model organism *C. elegans*.

## Conflict of Interest

The authors declare no conflict of interest.

## CRediT authorship contribution statement

**Fernando Calahorro:** Conceptualization, Data curation, Formal analysis, Investigation, Methodology, Validation, Visualization, Funding acquisition, Writing - original draft. **Lindy Holden-Dye:** Conceptualization, Funding acquisition, Methodology, Supervision, Writing - review & editing. **Vincent O’Connor:** Conceptualization, Funding acquisition, Methodology, Supervision, Writing - review & editing.

## References

[1] Hunt P.R. (2017). The C-elegans model in toxicity testing. J. Appl. Toxicol..

[2] Xiong H.J., Pears C., Woollard A. (2017). An enhanced C. elegans based platform for toxicity assessment. Sci. Rep..

[3] Hahn M.E., Sadler K.C. (2020). Casting a wide net: use of diverse model organisms to advance toxicology. Dis. Model. Mech..

[4] Lai C.H., Chou C.Y., Ch’ang L.Y., Liu C.S., Lin W.C. (2000). Identification of novel human genes evolutionarily conserved in Caenorhabditis elegans by comparative proteomics. Genome Res..

[5] Antoshechkin I., Sternberg P.W. (2007). The versatile worm: genetic and genomic resources for Caenorhabditis elegans research. Nat. Rev. Genet..

[6] Calahorro F., Ruiz-Rubio M. (2011). Caenorhabditis elegans as an experimental tool for the study of complex neurological diseases: Parkinson’s disease, Alzheimer’s disease and autism spectrum disorder. Invertebr. Neurosci..

[7] Chen X., Barclay J.W., Burgoyne R.D., Morgan A. (2015). Using C. elegans to discover therapeutic compounds for ageing-associated neurodegenerative diseases. Chem. Cent. J..

[8] Lucanic M., Garrett T., Yu I., Calahorro F., Shahmirzadi A.A., Miller A., Gill M.S., Hughes R.E., Holden-Dye L., Lithgow G.J. (2016). Chemical activation of a food deprivation signal extends lifespan. Aging Cell.

[9] Mathew M.D., Mathew N.D., Miller A., Simpson M., Au V., Garland S., Gestin M., Edgley M.L., Flibotte S., Balgi A., Chiang J., Giaever G., Dean P., Tung A., Roberge M., Roskelley C., Forge T., Nislow C., Moerman D. (2016). Using C. elegans forward and reverse genetics to identify new compounds with anthelmintic activity. PLoS Negl. Trop. Dis..

[10] Kwok T.C.Y., Ricker N., Fraser R., Chan A.W., Burns A., Stanley E.F., McCourt P., Cutler S.R., Roy P.J. (2006). A small-molecule screen in C-elegans yields a new calcium channel antagonist. Nature.

[11] Lehner B., Tischler J., Fraser A.G. (2006). RNAi screens in Caenorhabditis elegans in a 96-well liquid format and their application to the systematic identification of genetic interactions. Nat. Protoc..

[12] O’Reilly L.P., Long O.S., Cobanoglu M.C., Benson J.A., Luke C.J., Miedel M.T., Hale P., Perlmutter D.H., Bahar I., Silverman G.A., Pak S.C. (2014). A genome-wide RNAi screen identifies potential drug targets in a C. elegans model of alpha 1-antitrypsin deficiency. Hum. Mol. Genet..

[13] O’Reilly L.P., Luke C.J., Perlmutter D.H., Silverman G.A., Pak S.C. (2014). C. elegans in high-throughput drug discovery. Adv. Drug Deliv. Rev..

[14] O’Reilly L.P., Perlmutter D.H., Silverman G.A., Pak S.C. (2014). alpha l-Antitrypsin deficiency and the hepatocytes - an elegans solution to drug discovery. Int. J. Biochem. Cell Biol..

[15] Krajacic P., Shen X.N., Purohit P.K., Arratia P., Lamitina T. (2012). Biomechanical profiling of caenorhabditis elegans motility. Genetics.

[16] Buckingham S.D., Partridge F.A., Sattelle D.B. (2014). Automated, high-throughput, motility analysis in Caenorhabditis elegans and parasitic nematodes: Applications in the search for new anthelmintics. Int. J. Parasitol. Drugs Drug Resist..

[17] Izquierdo Patricia G., Christopher Green V.O.C., Holden-Dye Lindy, Tattersall John (2020). C. elegans pharyngeal pumping provides a whole organism bio-assay to investigate anti-cholinesterase intoxication and antidotes. Neurotoxicology.

[18] Scholz M., Lynch D.J., Lee K.S., Levine E., Biron D. (2016). A scalable method for automatically measuring pharyngeal pumping in C. elegans. J. Neurosci. Methods.

[19] Rodriguez-Palero M.J., Lopez-Diaz A., Marsac R., Gomes J.E., Olmedo M., Artal-Sanz M. (2018). An automated method for the analysis of food intake behaviour in Caenorhabditis elegans. Sci. Rep..

[20] Crisford A., Murray C., O’Connor V., Edwards R.J., Kruger N., Welz C., von Samson-Himmelstjerna G., Harder A., Walker R.J., Holden-Dye L. (2011). Selective toxicity of the anthelmintic emodepside revealed by heterologous expression of human KCNMA1 in Caenorhabditis elegans. Mol. Pharmacol..

[21] Leung M.C.K., Williams P.L., Benedetto A., Au C., Helmcke K.J., Aschner M., Meyer J.N. (2008). Caenorhabditis elegans: an emerging model in biomedical and environmental toxicology. Toxicol. Sci..

[22] Lockery S.R., Hulme S.E., Roberts W.M., Robinson K.J., Laromaine A., Lindsay T.H., Whitesides G.M., Weeks J.C. (2012). A microfluidic device for whole-animal drug screening using electrophysiological measures in the nematode C. elegans. Lab Chip.

[23] Weeks J.C., Robinson K.J., Lockery S.R., Roberts W.M. (2018). Anthelmintic drug actions in resistant and susceptible C-elegans revealed by electrophysiological recordings in a multichannel microfluidic device. Int. J. Parasitol. Drugs Drug Resist..

[24] Johnstone I.L. (2000). Cuticle collagen genes - expression in Caenorhabditis elegans. Trends Genet..

[25] Fritz J.A., Behm C.A. (2009). CUTI-1: a novel tetraspan protein involved in C. elegans CUTicle formation and epithelial integrity. PLoS One.

[26] Giacomotto J., Segalat L. (2010). High-throughput screening and small animal models, where are we?. Br. J. Pharmacol..

[27] Croll Neil A., Smith J.M. (1978). Integrated behaviour in the feeding phase of Caenorhabditis elegans (Nematoda). J. Zool..

[28] Goldstein P., Magnano L., Rojo J. (1992). Effects of dimethyl sulfone (Dmso2) on early gametogenesis in caenorhabditis-elegans - ultrastructural aberrations and loss of synaptonemal complexes from pachytene nuclei. Reprod. Toxicol..

[29] Sze J.Y., Victor M., Loer C., Shi Y., Ruvkun G. (2000). Food and metabolic signaling defects in a Caenorhabditis elegans serotonin-synthesis mutant. Nature.

[30] Chiang J.T., Steciuk M., Shtonda B., Avery L. (2006). Evolution of pharyngeal behaviors and neuronal functions in free-living soil nematodes. J. Exp. Biol..

[31] Hobson R.J., Hapiak V.M., Xiao H., Buehrer K.L., Komuniecki P.R., Komuniecki R.W. (2006). SER-7, a Caenorhabditis elegans 5-HT7-like receptor, is essential for the 5-HT stimulation of pharyngeal pumping and egg laying. Genetics.

[32] David Raizen B.-m.S., Trojanowski Nick, You Young-Jai, WormBook (2012). Methods for Measuring Pharyngeal Behaviors.

[33] Dillon J., Andrianakis I., Mould R., Ient B., Liu W., James C., O’Connor V., Holden-Dye L. (2013). Distinct molecular targets including SLO-1 and gap junctions are engaged across a continuum of ethanol concentrations in Caenorhabditis elegans. FASEB J..

[34] Frankowski H., Alavez S., Spilman P., Mark K.A., Nelson J.D., Mollahan P., Rao R.V., Chen S.F., Lithgow G.J., Ellerby H.M. (2013). Dimethyl sulfoxide and dimethyl formamide increase lifespan of C-elegans in liquid. Mech. Ageing Dev..

[35] Partridge F.A., Tearle A.W., Gravato-Nobre M.J., Schafer W.R., Hodgkin J. (2008). The C. elegans glycosyltransferase BUS-8 has two distinct and essential roles in epidermal morphogenesis. Dev. Biol..

[36] Calahorro F., Ruiz-Rubio M. (2012). Functional phenotypic rescue of Caenorhabditis elegans neuroligin-deficient mutants by the human and rat NLGN1 fenes. PLoS One.

[37] Hunter J.W., Mullen G.P., McManus J.R., Heatherly J.M., Duke A., Rand J.B. (2010). Neuroligin-deficient mutants of C. elegans have sensory processing deficits and are hypersensitive to oxidative stress and mercury toxicity. Dis. Model. Mech..

[38] Calahorro F., Keefe F., Dillon J., Holden-Dye L., O’Connor V. (2019). Neuroligin tuning of pharyngeal pumping reveals extrapharyngeal modulation of feeding in Caenorhabditis elegans. J. Exp. Biol..

[39] Law W.J., Wuescher L.M., Ortega A., Hapiak V.M., Komuniecki P.R., Komuniecki R. (2015). Heterologous expression in remodeled C. elegans: a platform for monoaminergic agonist identification and anthelmintic screening. PLoS Pathog..

[40] Castro C.A., Hogan J.B., Benson K.A., Shehata C.W., Landauer M.R. (1995). Behavioral-effects of vehicles - DMSO, ethanol, Tween-20, Tween-80, and emulphor-620. Pharmacol. Biochem. Behav..

[41] Maes J., Verlooy L., Buenafe O.E., de Witte P.A.M., Esguerra C.V., Crawford A.D. (2012). Evaluation of 14 organic solvents and carriers for screening applications in zebrafish embryos and larvae. PLoS One.

[42] Timm M., Saaby L., Moesby L., Hansen E.W. (2013). Considerations regarding use of solvents in in vitro cell based assays. Cytotechnology.

[43] Mitchell P., Mould R., Dillon J., Glautier S., Andrianakis I., James C., Pugh A., Holden-Dye L., O’Connor V. (2010). A differential role for neuropeptides in acute and chronic adaptive responses to alcohol: behavioural and genetic analysis in Caenorhabditis elegans. PLoS One.

[44] Davies A.G., Pierce-Shimomura J.T., Kim H., VanHoven M.K., Thiele T.R., Bonci A., Bargmann C.I., McIntire S.L. (2003). A central role of the BK potassium channel in behavioral responses to ethanol in C-elegans. Cell.

[45] Vrablik T.L., Petyuk V.A., Larson E.M., Smith R.D., Watts J.L. (2015). Lipidomic and proteomic analysis of Caenorhabditis elegans lipid droplets and identification of ACS-4 as a lipid droplet-associated protein. Biochim. Biophys. Acta.

[46] Smus J.P., Ludlow E., Dalliere N., Luedtke S., Monfort T., Lilley C., Urwin P., Walker R.J., O’Connor V., Holden-Dye L., Mahajan S. (2017). Coherent anti-Stokes Raman scattering (CARS) spectroscopy in Caenorhabditis elegans and Globodera pallida: evidence for an ivermectin-activated decrease in lipid stores. Pest Manag. Sci..

[47] Arvanitis M., Li D.D., Lee K., Mylonakis E. (2013). Apoptosis in C. elegans: lessons for cancer and immunity. Front. Cell. Infect. Microbiol..

[48] Rajasekharan S.K., Lee J. (2020). Hydropic anthelmintics against parasitic nematodes. PLoS Pathog..

[49] Richmond J.E., Jorgensen E.M. (1999). One GABA and two acetylcholine receptors function at the C. elegans neuromuscular junction. Nature Neurosc ..

[50] Brenner S. (1974). The genetics of Caenorhabditis elegans. Genetics.

