# Supplementary material for: Impact of drug solvents on C. elegans pharyngeal pumping
Source: Toxicol Rep. 2021 Jun 17;8:1240–7. doi: 10.1016/j.toxrep.2021.06.007 (PMC8233170; doi:10.1016/j.toxrep.2021.06.007)
Supplement: Supplementary file 1 [file mmc1.docx]

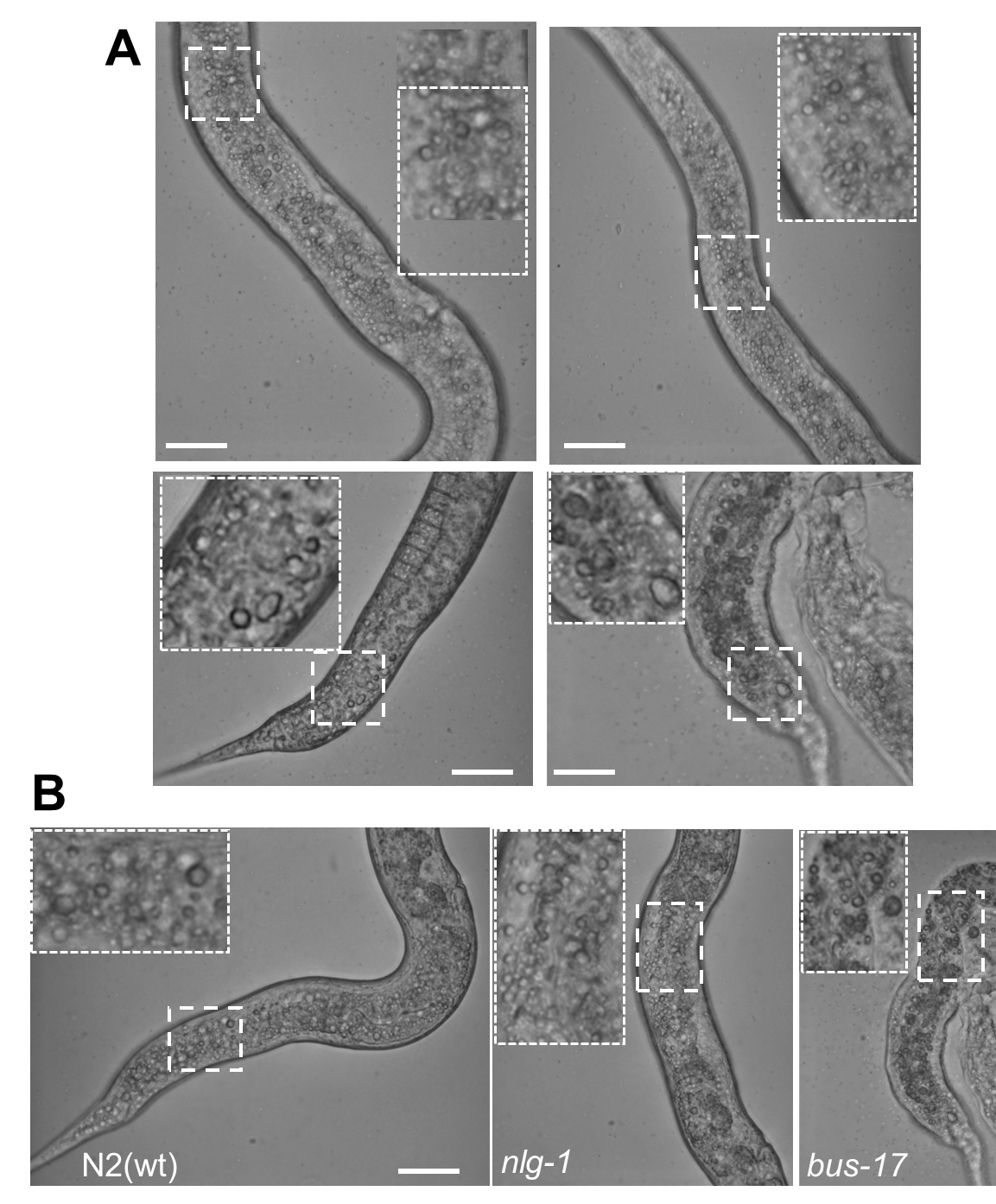


**Supplement to Figure 6. Accumulation of internal membrane-like structures in animals after chronic exposure to DMSO**

**A.** Independent images of individual N2 (wt) animals exposure to 1% (v/v) DMSO for 3hrs showing the consistent accumulation of internal membrane-like structures within the pseudocoelom surrounding the pericellular structures. **B.** The appearance of these internal membrane-like structures are sustained with no clearance after 24 hrs of chronic exposure to DMSO, and they are persistent up to 24 hrs after removal from DMSO in N2 (wt), *nlg-1* and *bus-17* animals.

The boxes inset in each image correspond to the corresponding magnified area highlighted with dashed boxes in the worm’s body. Scale bars represent 100 μm.
